# Oral health status of patients with acute coronary syndrome – a case control study

**DOI:** 10.1186/1472-6831-12-17

**Published:** 2012-06-22

**Authors:** Dirk Ziebolz, Andrea Priegnitz, Gerd Hasenfuß, Hans-Joachim Helms, Else Hornecker, Rainer F Mausberg

**Affiliations:** 1 Department of Preventive Dentistry, Periodontology and Cariology, University Medical Centre Goettingen, Goettingen, Germany; 2 Department of Cardiology and Pneumology, University Medical Centre Goettingen, Goettingen, Germany; 3 Department of Medical Statistics, University Medical Centre Goettingen, Goettingen, Germany

**Keywords:** Oral health, Oral hygiene, Gingival inflammation, Periodontitis, Acute coronary syndrome, Acute myocardial infarction, Unstable angina pectoris

## Abstract

**Background:**

The aim of this investigation was to assess the state of oral health of patients with acute coronary syndrome (ACS) and to compare this with that of a provably healthy control group (H).

**Methods:**

33 patients who were receiving treatment as inpatients following acute myocardial infarction or unstable angina pectoris took part in the study (ACS-group). A healthy control group (H-group) made up of blood donors, was formed following matching for age, gender, and smoking habit with the study patient group.

The dental investigation consisted of the dental status (DMF-T), a plaque-Index (PI), an assessment of gingival inflammation (GI) and periodontal situation (Periodontal Screening Index: PSR®/PSI), and attachment loss (AL). Statistical evaluation: t-test, Mann–Whitney-test and chi- squared test (level of significance p < 0.05).

**Results:**

The mean DMF-T of the ACS-group (18.7 ± 6.8) and the H-group (19.4 ± 5.1) showed no difference (p = 0.7). Although, in the ACS-group the average loss of teeth (M-T: 8.4 ± 5.2) was higher than in the H-group (M-T: 5.8 ± 6.6) the difference was not significant (p = 0.2). Whereas with the PI no difference between the two groups was found (p = 0.9), the ACS-group showed significantly more signs of inflammation (GI) than the H-group (p = 0.045). In the case of PSR®/PSI, there was no difference between the two groups (p = 0.7). With regard to AL, no difference was revealed between ACS- and H-group (p = 0.2).

**Conclusion:**

Although, the state of oral health of the ACS-group differed only insignificantly from that of control, patients with ACS showed more signs of gingival inflammation and a higher loss of teeth.

## Background

Worldwide, coronary heart disease (CHD) represents the most frequent disease and cause of death. The prevalence in men between the age of 45 to 54 years is 2-5 %, and that of the age of 65 to 74 years is 11-20 % [1] .Moreover, the annual rate of incidence of coronary events in Germany lies between 78 (for women) and 370 new illnesses (for men) per 100.000 inhabitants [2]. In addition to stable cardiovascular diseases, acute and therefore life-threatening forms such as unstable angina pectoris (AP) and acute myocardial infarction (AMI), are of particular importance. They are grouped together by the term a*cute coronary syndrome* (ACS) [3].

A number of different risk factors, for example, hyperlipoproteinemia, diabetes mellitus, high blood pressure, smoking, and age, as well as male gender, predispose individuals to CHD [4-6]. In addition, bacterial and viral infections have been reported to be aetiological risk factors for cardiovascular diseases [7,8]. Amongst other factors, inflammatory diseases of the periodontium, such as periodontal diseases (PD), i.e. gingivitis and periodontitis, also come under consideration [9].The primary cause of PD is microbial infestation of oral cavity with a complex biofilm of various periodontal pathogenic bacteria [10].The aetiology and pathogenesis of PD, however, is multi-factorial and negatively influenced by a number of risk factors, such as smoking and stress, but also systemic diseases such as, e. g., diabetes mellitus [11-14]. Consequently, both inflammatory PD and CHD are chronic diseases with similar aetiological factors. Moreover, there is increasing evidence that poor oral hygiene and especially the presence of inflammatory PD increase the risk of CHD. On the basis of a number of studies, an association between chronic cardiovascular diseases (arteriosclerosis) and PD is likely [15-18]. There have also been an increasing number of reports of an association between periodontal inflammation and AMI [19-25]. However, the results of some other investigations have indicated that there is no association between CHD (and/or AMI) and PD [26-29].

The discrepancy between the results is possibly due to the inhomogeneity of implementation (study design, sample composition) of the different studies. Also, on the one hand, different diagnostic criteria for PD were used, and, on the other, in a number of investigations, there was an unequal distribution and inclusion of patients with different cardiovascular risk factors, such as systemic diseases (diabetes mellitus) and smoking habits compared to the control group. This may have influenced the results as a possible confounder [30,31]. In addition, details about the composition of the control groups in terms of general state of health were inadequate.

On the basis of the current state of the literature, further assessment of a possible association between the state of oral health, including PD as well as tooth loss and caries experience, and ACS compared to an established healthy control group is important. This could contribute to a clarification of the relationship between oral health and ACS. The aim of the investigation was therefore to evaluate the state of oral health in patients with ACS, i.e. AMI or unstable angina pectoris, and to compare this with that of a healthy control group (H).

## Methods

In this case control study, the oral health status of patients with ACS (AMI or AP) was compared with healthy individuals. The study was reviewed and approved by the ethics committee of the University Medical Centre Goettingen, Germany (No. 5/2/03).

### Participants

#### Acute coronary syndrome group (ACS-group)

In-patients who were treated in the Department of Cardiology and Pneumology of the University Medical Centre Goettingen, because of ACS were asked to participate in the study on a voluntary basis. Patients were included following written informed consent. The corresponding diagnosis was made by the cardiologists on the basis of the anamnesis, changes in Electro-Cardio-Gramm: ECG (ST section elevation, T-inversion), laboratory analyses of the patients´ serum (Creatine-Proteine-Kinase (CPK), Troponin T) and/or according to the coronary angiography, following the guidelines of the European Society of Cardiology (ESC) [3]. The following inclusion criteria were a prerequisite for participation in the study: a stable physical and psychological condition, allowing to carry out a dental examination within three to five days following admission as an inpatient, no risk of endocarditis, and aged no older than 68 years. Because of potential interactions, the following exclusion criteria were defined: other medical disorders, such as diabetes mellitus, acute-inflammatory diseases, infectious diseases (hepatitis, HIV), osteoporosis and tumour diseases, alcohol abuse, as well as long-term drug therapy with phenytoine, nifedipine and cyclosporine.

As smoking affects both cardiovascular and periodontal diseases, the patients were asked about their nicotine status already on the occasion of making the first contact with medical staff.

#### Healthy group (H-group)

As a control, individuals who were proven healthy and who regularly attended the Transfusion Medicine Department of the University Medical Centre Goettingen for blood-donation were asked to participate in the study (Note: In Germany a comprehensive medical check up is mandatory to verify the subjects` health before blood-donation). At the same time, the control subjects were required to match the cardiology patients in terms of age (max. ± 2 years) and gender. Accordingly, the smoking habits of the patients and the comparison subjects were required to be similar. Matching procedure in summary: matching one to one in terms of age, gender and smoking habits. Inclusion and exclusion criteria were analogous to the ACS-group.

### Questionnaire

A questionnaire covered the following themes:

A. **Oral hygiene behavior (good / unsatisfactory)** for this, the following information was requested: the number of tooth-brushing each day, when this took place (in relation to mealtimes), and for how many minutes the teeth were brushed on each occasion. Based on the Fourth German Oral Health Study (DMS IV), conclusions were drawn regarding individual oral hygiene behavior, enabling this to be classified as “good oral hygiene behavior” according to the following criteria: [32]

Oral hygiene measures being carried out at least twice a day,

Oral hygiene measures being carried out after a meal or before going to bed

Brushing the teeth for at least two minutes.

B Making use of the provision of dental care or treatment *(“control-oriented” or ”complaint- oriented” behavior)* this was evaluated by asking how long ago the last visit to the dentist took place, the reason for the visit, and how often the dentist was visited during one year.

C. Possible reasons for the loss of teeth were listed the participants were required to mark the relevant reasons in general and in relation to their teeth.

### Dental examination

All subjects were examined once under standardized conditions by an experienced dentist (AP) in the dental clinic of the University Medical Centre Goettingen. The dental examination included the dental status (DMF-T), assessment of oral hygiene / gingival inflammation (PI, GI) and the evaluation of the periodontal situation (Periodontal Screening Index: PSR®/PSI, attachment loss: AL). The ACS patients were examined within 3–5 days after the cardiac event and the healthy control patients received a fixed appointment for the examination.

#### Dental status (DMF-T)

The DMF-T was assessed visually with mirror and probe. All teeth with a reasonable suspicion of/or definitely showing a cavity in the dentine layer were assigned to the D (= decayed) category. Filled and crowned teeth were evaluated category F (=filled); missing teeth were assigned to the M (=missing) category.

The degree of caries restoration (%) was calculated: ratio of filled teeth (F-T) to the carious (D-T) plus filled teeth (F-T) (F-T/(D-T + F-T) x 100).

#### Oral hygiene findings (PI, GI)

[33,34]: The plaque-index (PI) visually assesses the accumulation of plaque (without dyeing the plaque) in terms of four categories (0 = no plaque to score 3 = a lot of plaque on the teeth and along the gingival margin). Each tooth was evaluated at four points (mesial, buccal, distal, oral) and the highest score determined.

In addition, gingival inflammation was assessed visually with the gingiva index (GI) using 4 scores (0 = free from inflammation to score 3 = severe inflammation, strong reddening, a tendency to spontaneous bleeding and ulceration); in this case, as well, each tooth was assessed at four points and the highest score was determined.

#### Periodontal situation (PSR®/PSI, AL)

The periodontal situation was evaluated with the periodontal screening index (PSR®/PSI: “Periodontal Screening & Recording – PSR®/ “Periodontal Screening Index - PSI” in Germany) [35, 36], and attachment loss (AL) [37]. The examination was performed with the WHO probe (Morita, Kyoto, Japan) at 6 points per tooth (mesio-buccal, buccal, disto-buccal, disto-oral, oral, mesio-oral). The PSR®/PSI scores (0 to 4) were recorded: score 0 = healthy, score 1 = bleeding, score 2 = supra-/subgingival dental calculus, score 3 = probing depths from 3.5 mm to max. 5.5 mm, score 4 = probing depths greater than 5.5 mm. For each sextant, the highest PSR®/PSI score was determined and the overall largest PSR®/PSI value established the periodontal situation [38].

The loss of attachment (AL) assessed the extent of loss of periodontal support tissue, i.e. the distance from the cemento-enamel-junction (CEJ) to the pocket fundus. The AL was determined using five scores: 0 = CEJ not visible/PSI < score 4, 1 = AL 4-5 mm, 2 = AL 6-8 mm, 3 = AL 9-11 mm, 4 = AL > 12 mm. In this case, as well, for each sextant the highest score was determined.

### Statistical evaluation

All data were entered and controlled twice by two independent investigators. The statistical evaluation was carried out using the statistics program STATISTICA (Version 6.1).

Data was assessed for normal distribution. Accordingly, Student’s t-test was used if normal distribution could be assumed and Mann–Whitney-U test otherwise. Chi-square test was used for dichotomous data. In this analysis, the parameters: D-T, M-T, F-T, degree of caries restoration, plaque index (PI) and gingivitis index (GI) were analysed using the Mann–Whitney test and for the parameters age and DMF-T an unpaired T-test were used. In order to evaluate the questionnaires regarding oral hygiene behaviour, reason for tooth loss, tooth loss, PSR®/PSI and AL were compared using the chi- squared test. The level of significance was set at p < 0.05.

## Results

### Participants

#### Acute coronary syndrome group (ACS-group)

Of the total of 49 consecutive patients documented for the period of the study, 33 (m = 30; f = 3) could be included in the investigation following the inclusion and exclusion criteria. All 33 patients were treated because of ASC: 17 (52 %) because of an acute myocardial infarction and 16 (48 %) because of an unstable angina pectoris. The average age was 52.8 years (min. 34, max. 68 years). With regard to smoking 15 were current smokers, 11 former smokers and 7 non-smokers (Table 1).

**Table 1:** Matching characteristic of the participants of both groups

		**ACS-group**[33]		**H-group**[33]
		**AMI**[17]	**AP**[16]	
**gender (male)**		30		30
		16	14	
**age (mean years ± SD, range )**		52.8±8.7 (34 – 68)		53.1±8.8(35 – 67)
		51.0±8.6	54.5±7.7	
**smoking habits**	**non smoker**	7		7
	**former smoker**	11		11
	**smoker**	15		15
**serum CRP in mg/l (mean ± SD)**		6.9±7.18		2.05±0.25
**medication**	**ASS**	22		0
	**Clopidogrel** (Isocover®)	17		0
	**Metoprolol** (Beloc Zok®)	20		0
	**x1Pravastatin** (Pravasin®)	7		0

(ACS-group: acute coronary syndrome, AMI: acute myocardial infarction, AP: angina pectoris,H-group: healthy control, CRP: c-reactive protein, [n])

#### Healthy group (H-group)

According to the matching criteria 33 blood donors (m = 30; f = 3) were included as controls for the study. The average age of the subjects was 53.1 years (min. 35, max. 67 years). With regard to smoking 15 were current smokers, 11 former smokers and 7 non-smokers (Table 1).

### Questionnaire

#### A: Oral hygiene behavior

The majority of participants had good oral hygiene behavior (ACS: n = 20 (61 %), H: n = 23 (70 %)). The difference between the two groups was not significant (p = 0.8).

#### B: Making use of dental care or dental treatment

In each of the two groups, there were 16 participants (48 %) who had received dental treatment in the past three months, as well a further nine ACS patients (27 %) and eight H subjects (24 %) had received dental treatment in the past 3–6 months. Regarding the remaining participants, the last visit to the dentist was much longer ago. The difference between the groups was not significant (p = 0.6). The majority of the ACS patients and H subjects showed controlled behavior and visited routinely a dentist once or twice a year. Six participants in the ACS-group (18 %) and four in the H-group (12 %) showed complaint-oriented behavior. The difference between the groups was not significant (p = 0.3).

#### C: Reasons for tooth loss

For both groups, it was not possible to determine the reasons (tooth-related reasons) for loss of teeth completely, but the main reason for tooth loss was attributed to destruction by caries (Table 2).

**Table 2:** Number of/reasons for tooth loss: regarding the subject and the teeth

			**ACS-group**[33]	**H-group**[33]	**statistical analysis** (test)	**level of significance** (p-value)
regarding the subject	no tooth loss	all	16	13	Chi-square test	n.s.
		maxilla	9	8		n.s.
		mandibula	7	5		n.s.
	reason for tooth loss	caries	19	28	Chi-square test	n.s.
		periodontitis	5	0		n.s.
		other	2	3		n.s.
		unknown	11	9		n.s.
		various reasons	13	13		n.s.
regarding the teeth	number of tooth loss	all	278	190	Chi-square test	<0.0001
		maxilla	150	106		0.0006
		mandibula	128	84		0.0012
	reason for tooth loss	caries	140	75	Chi-square test	n.s
		periodontitis	61	3		<0.001
		other	20	26		n.s.
		unknown	57	86		0.0001

(ACS-group: acute coronary syndrome, H-group: healthy control, [n], n.s.: not significant = p > 0.05).

#### Dental status (DMF-T)

The mean DMF-T of the ACS-group was 18.7 ± 6.8, and that of the H-group 19.4 ± 5.1 (Table 3). The difference was not significant (p = 0.6). Regarding the components D-T and F-T no significant difference between the two groups could be observed (D-T: p = 0.1; F-T: p = 0.2; Table 3). The mean M-T of the ACS-group (8.4 ± 5.2) was clearly higher than the mean M-T of the H-group (5.8 ± 6.6; Table 3), nevertheless, the difference was not significant (p = 0.2; Table 3).

**Table 3:** Comparison of dental and oral health parameters of participants in both groups

		**ACS-group**[33]	**H-group**[33]	**statistical analysis** (test)	**level of significance** (p-value)
**DMF-T (mean ± SD)**		18.7 ± 6.8	19.4 ± 5.1	t-test	n.s
**D-T (median, range)**		1 (0–17)	3 (0–13)	Mann–Whitney-U test	n.s.
**M-T (median, range)**		4 (0–28)	4 (0–28)	Mann–Whitney-U test	n.s.
**F-T (median, range)**		9 (0–20)	11 (0–23)	Mann–Whitney-U test	n.s
**degree of caries restoration (median, range)**		91 % (11-100 %)	80 % (24-100 %)	Mann–Whitney-U test	0.046
**caries treatment need (median, range)**		9 % (89-0 %)	20 % (76-0 %)	Mann–Whitney-U test	n.s.
**oral hygiene parameters**	**PI (median, range)**	0.75 (0–3)	0 (0–3)	Mann–Whitney-U test	n.s
	**GI (median, range)**	1 (0–3)	0 (0–2)	Mann–Whitney-U test	0.045

(ACS-group: acute coronary syndrome, H-group: healthy control, DMF-T: number of decayed, missing and filled teeth (caries index), D-T: decayed teeth, M-T: missing teeth, F-T: filled teeth, PI: Plaque-Index and GI: Gingiva-Index, SD: standard deviation, n.s.: not significant = p > 0.05).

Regarding the total number of tooth loss the difference between the ACS-group and the H-group was significant (p < 0.0001; Table 2). In each group, there was one edentulous participant.

The ACS-group showed a significantly higher level of caries restoration than the H-group (p = 0.046). Accordingly, the caries treatment need was higher in the H-group (Table 3).

#### Oral hygiene findings (PI, GI)

The median PI of the ACS-group was 0.75, that of the H-group 0, the difference was not significant (p = 0.9) (Table 3).

The ACS-group showed significantly more signs of gingival inflammation (GI: median 1), than the H-group (GI: median 0; p = 0.045; Table 3).

#### Periodontal situation (PSR®/PSI, AL)

Four ACS patients (12 %) and one H subject (3 %) were periodontally healthy (PSR®/PSI score 0). One participant in each group (3 %) showed bleeding on probing (PSR®/PSI score 1), and PSR®/PSI score 2 was found in six patients of the ACS-group (18 %) and 12 H subjects (36 %). PSR®/PSI score 3 or 4 was found in 16 (48 %) and five ACS patients (15 %), respectively, and in 13 (39 %) and five H subjects (15 %), respectively. In summary, almost two thirds of ACS patients (64 %) and about half of H subjects (55 %) showed “moderate to severe periodontitis” (PSR®/PSI score 3/4), and seven of ACS patients (21 %) and 13 of H subjects (39 %) showed signs of “gingival inflammation” (PSR®/PSI score 1/2). The difference between the two groups was not significant (p = 0.7; Table 4).

**Table 4:** Description of the result of periodontal parameters (PSR®/PSI and AL) of participants in both groups

		**ACS-group**[33]		**H-group**[33]	
		number of patients [33]	number of sextants [198]	number of patients [33]	number of sextants [198
PSR®/PSI max	score 0	4 (12 %)	43 (22 %)	1 (3 %)	67 (34 %)
	score 1	1 (3 %)	21 (11 %)	1 (3 %)	21 (11 %)
	score 2	6 (18 %)	46 (23 %)	12 (36 %)	48 (24 %)
	score 3	16 (48 %)	48 (24 %)	13 (39 %)	44 (22 %)
	score 4	5 (15 %)	9 (5 %)	5 (15 %)	6 (3 %)
	X	1 (3 %)	31 (16 %)	1 (3 %)	12 (6 %)
AL max	AL 0	16 (48 %)	117 (59 %)	13 (39 %)	137 (69 %)
	AL 1	7 (21 %)	22 (11 %)	10 (30 %)	35 (18 %)
	AL 2	9 (27 %)	21 (11 %)	7 (21 %)	12 (6 %)
	AL 3	0	0	2 (6 %)	2 (6 %)
	AL 4	0	0	0	0
	X	1 (9 %)	31 (16 %)	1 (3 %)	12 (6 %)

In the ACS-group, AL 0 (no loss of attachment) was observed more frequently than in the H-group (ACS: n = 16 / 48 %; H: n = 13 / 39 %). The highest AL of 1 was found in seven ACS patients (21 %) and 10 H subjects (30 %); nine ACS patients (27 %) and seven H subjects (21 %) had an AL 2; and an AL of 3 was observed in two H subjects (6 %) and no ACS patient. The difference between the groups was not significant (p = 0.2; Table 4).

## Discussion

In this study 33 patients with acute coronary syndrome (ACS-group) were matched in terms of age, gender, and smoking behavior with a proven generally healthy group of blood donors (H-group) and studied with regard to their oral health status. The majority of participants in both groups showed good oral hygiene behavior and control-oriented behavior in relation to the use of dental care and treatment. For the parameter DMF-T, no difference was found between the two groups. Although, in the ACS-group the average loss of teeth (M-T) was higher than in the H-group, the difference was not significant. Regarding the total number of tooth loss the difference between the ACS-group and the H-group was significant. In relation to oral hygiene status (PI) no difference was found between the two groups. In contrast, the ACS patients showed significantly more signs of gingival inflammation (GI) than the H subjects. In the assessment of the periodontal status (PSR®/PSI as well as AL), there were no differences between the groups. Nevertheless, two-third of the ACS-group and approximately one half of the H-group had moderate to severe periodontitis, a finding which was confirmed by the AL.

The finding that the ACS patients showed a higher rate of tooth loss than the H subjects confirmed some previous studies [39-41]. Other studies were unable to find a significant difference in the number of missing teeth [19,20,22,24]. Moreover, a large prospective study in Sweden showed a close relationship between number of missing teeth and cardiovascular and coronary heart diseases (and even cardiovascular mortality), indicating a link between coronary artery disease and oral health [42]. One participant of each group was toothless. In other studies, the number of toothless individuals with ACS was higher (6–35 %) [40,43].

In this study the reasons for tooth loss could not be definitely determined but was mainly attributed to destruction by caries. Only in 22 % of the cases periodontitis was given as the reason for tooth loss.

A comparison of the DMF-T of the ACS patients investigated in this study with other studies in CHD patients is limited by the fact that only the number of missing teeth and/or the number of remaining teeth were given. However, the results for DMF-T in the present study (ACS: 18.7 ± 6.8; H: 19.4 ± 5.1) are between the findings for the age group 35 to 44 years (DMF-T = 14.5) and age group 65 to 74 years (DMF-T = 22.1) in the DMS IV (German Study on Oral Health, part IV) [32].

Considering the results for PI the differences between the groups were not significant, but oral hygiene in the ACS-group tended to be worse. This finding has been obtained by two other studies [24,44]. In the case of ACS patients, however, gingival inflammation was increased significantly versus H subjects. It should be taken into account that 18 % of the ACS patients in the studies mentioned above had diabetes mellitus[44], and in addition, there were differences in smoking behavior between the ACS patients and the control subjects [24,44]. This could possibly have influenced (confounder) the results. In the present study these factors were taken into account by matching procedure and exclusion criteria, and can be excluded as a possible confounder.

Other research groups used other indices for assessing the state of gingival inflammation, e. g., bleeding on probing (BOP); they observed that the ACS patients showed a worse gingival status than control subjects [40,43]. These findings could be confirmed by the results of this study. Only one investigation established no difference between the groups [20].

In the present study no difference was found between ACS-group and H-group with regard to the periodontal situation (PSR®/PSI and AL). However, two-third of the ACS patients had an increased periodontal treatment need, despite regular visits to the dentist. In contrast, Willershausen et al.[41] found a significant higher amount of PSI score 4 in their ACS patients, however, it is to mention that there were clearly (significantly) more smokers and diabetics in their ACS-group than in the corresponding control group. Whereas, Katz et al.[45] were unable to find a significant difference, but instead a tendency of a correlation between CPITN 4 in CHD patients. An association between moderate periodontitis and cardiac disease was likewise not found by this research group [45]. Spahr et al.[46], however, reported higher amount of CPITN values (3 and 4) in their CHD patients than in controls. Moreover, a number of authors observed deeper (probing) pocket depths in CHD/ACS patients than in corresponding controls [20,24,39,40,43,44]. The millimetre measurements ranged between 1.8 mm and 5 mm [39,40]. Converted into PSR®/PSI, these values correspond to the scores 0 to 3 and reflect rather moderate periodontitis. In contrast to this, some studies have indicated that in ACS patients significantly more measurement points with increased probe depths of >4 mm or >5 mm were present than in control subjects [19,22]. These findings correspond to PSI scores 3 and 4 and are comparable with the results presented here.

Likewise, in the present study, no statistically significant difference was found in relation to loss of attachment (AL). In contrast to this, other studies showed a significant correlation between CHD/ACS and an increased loss of attachment [24,39]. Thus, an association between a high loss of attachment and CHD/ACS diagnosis with an OR of 1.5 or 3.8, respectively, has been reported [21,47].

A number of authors established a correlation between a poor state of oral hygiene and CHD/ACS [20-22,24,25,39-45,47]; according to a meta-analysis, there is an OR of 2.35 for ACS with poor oral hygiene [23]. Other authors reported there is no association between oral hygiene and CHD/ACS [26-29]. This largely agrees with the results of this study. Although the ACS patients in this study had poor oral hygiene, the differences in relation to the healthy control group were marginal. Only in relation to signs of gingival inflammation there were significant differences.

Limitation of the study: Only 33 patients/subjects were included per group. However, they were carefully controlled for confounder such as age and smoking behavior. In addition, for the first time in a case control study, a control group with healthy subjects confirmed by a physician was included. PSR®/PSI was used to assess the periodontal situation. This index provides sufficient information on the condition of the periodontium and allows a comprehensive evaluation of the periodontal situation [35,36]. However, it is a screening index to assess the periodontal treatment need. In contrast, there is no information on the actual situation regarding the periodontal destruction. For this reason, AL was used additionally, in order to provide meaningful results. However, it should be noted that, in the pathogenesis of PD and with regard to the consequences for the general state of health, the composition of the biofilm (microbial plaque) plays a decisive role. This point was not taken into account in the study presented here.

## Conclusions

Although, the state of oral health of the ACS-group did not differ significantly from that of controls, patients with ACS showed more signs of gingival inflammation and a higher number of tooth loss. In order to clarify the causal relationship between oral and cardiovascular diseases (CHD/ACS), further studies are necessary.

## Competing interests

The authors declare that they have no competing interests.

## Authors' contributions

DZ interpreted the data and wrote the manuscript. AP carried out the clinical examination. GH has been involved in revising the manuscript critically for important intellectual content and has given final approval of the version to be published. HJH performed the statistical analysis. EH conceived of the study, and participated in its design and coordination, and has been involved in drafting the manuscript. RM was the head of the study and has made substantial contributions to conception and design. All authors read and approved the final manuscript.

## Pre-publication history

The pre-publication history for this paper can be accessed here:

http://www.biomedcentral.com/1472-6831/12/17/prepub
